# Vermont

**Published:** 1896-04

**Authors:** 


*Vermont.* Mr. Victor I. Spear, member of the Cattle Commision, speaking on the subject of "Disinfection of Stables" as an aid to the eradication of tuberculosis, said that the free use of a solution of bichloride of mercury over all the woodwork, after the latter had been thoroughly swept down, care being taken that no pools be left where cattle might lick the same; the use of live steam where the stable might be closed up sufficiently tight to maintain the high temperature of steam, and, as another valuable assistant, the use of sulphur fumes after closing up the barn thoroughly tight, would all prove effectual methods. Referring to the existence of tuberculosis in his own State, he said that he believed it could be thoroughly and completely eradicated, that it had been transplanted there by the introduction of diseased cattle from time to time, and had never existed there to the extent supposed or for the time some claimed; if it had, he believed that there would not be to-day a herd left in the State free from the disease.

